# Are Plastic Surgery Trainees Accurate Assessors of Their Own Microsurgical Skill?

**DOI:** 10.1016/j.jpra.2023.04.004

**Published:** 2023-04-27

**Authors:** David Carolan, Robert Milling, Christine Quinlan, Shane Carr, Anna Kinsella, Bronwyn Reid McDermott, Alan Hussey, Shirley Potter

**Affiliations:** 1 School of Medicine, University College Dublin, Belfield, Dublin 4; 2 Department of Plastic and Reconstructive Surgery, Mater Misericordiae University Hospital, Eccles St, Dublin 7; 3 The Pillar Centre for Transformative Healthcare, Mater Misericordiae University Hospital, Eccles St, Dublin 7; 4 Department of Plastic and Reconstructive Surgery, Galway University Hospital, Newcastle Road, Galway; 5 Irish Centre for Applied Patient Safety and Simulation, Galway University Hospital, Newcastle Road, Galway

**Keywords:** Microsurgery, Plastic and Reconstructive Surgery, Simulation, Self-assessment

## Abstract

**Background:**

Microsurgery is a technically demanding surgical discipline with a steep learning curve. Trainees have faced several difficulties due to less hands-on theater time and pandemic-related limits on access to technical training. To overcome this, trainees engaged in self-directed training, which requires an accurate self-assessment of skill. This study aimed to assess the ability of trainees to accurately self-assess their performance while performing a simulated microvascular anastomosis.

**Methods:**

Novice and specialist plastic surgery trainees performed a simulated microvascular anastomosis on a high-fidelity chicken femoral vessel model. Each participant objectively rated the quality of their anastomosis using the Anastomosis Lapse Index (ALI). Two expert microsurgeons subsequently blindly rated each anastomosis. To determine the accuracy of self-evaluation, self-scores and expert-scores were compared using a Wilcoxon signed-rank test.

**Results:**

Twenty-seven surgical trainees completed the simulation, with a mean time to completion (TTC) of 40.3 minutes (range 14.2–106.0 minutes). For the entire cohort, the median ALI self-score was 4 (range 3–10), while the median ALI expert-score was 5.5 (range 2.5–9.5). There was a significant difference between the ALI self-score and the expert-score (p<0.001). When grouped by experience level, there was no significant difference between self-score and expert-score within the specialist group, while there was a significant difference within the novice group (p=0.001).

**Conclusion:**

These findings suggest that specialist trainees are accurate assessors of their own microsurgical skills, but novice trainees tend to overestimate their technical skills. While novice trainees can engage in independent self-directed microsurgical training, they should seek expert feedback to ensure targeted training.

## Introduction

Microsurgery combines magnification, precision tools, and intricate operative techniques to anastomose small blood vessels, enabling the complex repair of human tissue after trauma, cancer, and congenital deficiencies. Over recent decades, it has evolved to become a routine part of Plastic and Reconstructive Surgery practice. Microsurgery requires significant training to attain proficiency. A microvascular anastomosis requires the surgeon to operate on vessels with a caliber of 1–2 mm in diameter under stereoscopic vision with minimal haptic feedback or dimensional perspective.^1^^,^^2^ Alongside the technical complexity of microsurgery, the procedures are often high-risk, presenting trainees with a steep and unforgiving learning curve.^3^^,^^4^

In the past, microsurgical education followed the traditional Halstedian training model, which relied upon the surgical trainee receiving graded exposure to performing microsurgery in theater.^5^^,^^6^ In recent years, there has been a transition toward simulation-based and independent, self-directed training for early skill acquisition.^7^^,^^8^ This has become increasingly important with recent changes to public policy and the COVID-19 pandemic enforcing restrictions on trainee working hours and theater teaching time. Consequently, there is a need for surgical trainees to engage in self-directed training to optimize the use of healthcare resources.

As simulation training grows in popularity, several assessment tools have been developed to assess microsurgical competency.^9^, ^10^, ^11^, ^12^, ^13^, ^14^, ^15^, ^16^, ^17^, ^18^ The Anastomosis Lapse Index (ALI) is a widely used microsurgical assessment tool that allows an assessor to objectively evaluate the quality of a trainee's microsurgical skill by identifying 10 commonly observed technical errors from a pre-determined list.^9^ Each error observed during anastomosis earns a score of 1 point, with the final score ranging from 0 to 10. Higher scores indicate a lower quality of anastomosis, while a score of 0 represents flawless anastomosis. The ALI provides a reference range for interpreting scores, suggesting that experts achieve a score below 3, while intermediate trainees attain a score between 3 and 6, and novice trainees typically score above 6. Figure 2 presents a copy of the scoring system. It is simple to use and is both time- and cost-efficient, requiring little additional resources. Previous studies have demonstrated the ALI to have both construct and predictive validity, as well as the ability to effectively identify improvements in technical skill following formal microsurgical training when the assessment was carried out by an independent assessor.^9^ However, no previous studies have evaluated whether it is an effective tool for self-assessment of microsurgical skills.

For independent training to effectively translate to improved microsurgical technical competency, trainees must be adept at accurate self-assessment of their technical skills. Previous studies have explored the ability of surgical trainees to self-assess their technical skills when performing laparoscopy, endoscopy, and knot-tying procedures, with inconclusive results.^19^, ^20^, ^21^, ^22^, ^23^, ^24^ There is a lack of research exploring whether trainees can accurately self-assess their microsurgical skills.

This study aimed to evaluate the ability of trainees to self-assess their technical skills when performing a simulated microvascular anastomosis. Secondly, we aimed to determine whether experience or gender influenced self-assessment accuracy or self-perceived technical skill.

## Materials/Patients and Methods

This study was conducted across two sites as part of a multicenter trial. Ethical approval was granted by the Mater Misericordiae University Hospital (MMUH) ethics committee and the Galway University Hospital (GUH) ethics committee. Data reporting followed the Strengthening the Reporting for Observational Studies in Epidemiology guidelines.

The study recruited novice and experienced plastic surgery trainees from MMUH and GUH, and each participant was given a participant information booklet outlining the study objectives and the testing procedure. All participants provided written informed consent before inclusion in the study. All participants completed a short demographics questionnaire detailing their gender, stage of training (Senior House Officer/Registrar/Specialist Registrar), and microsurgical experience. Following that, trainees were divided into groups based on their prior exposure to microsurgery using the criteria Tang et al.^25^ previously outlined.

**Figure 1:**
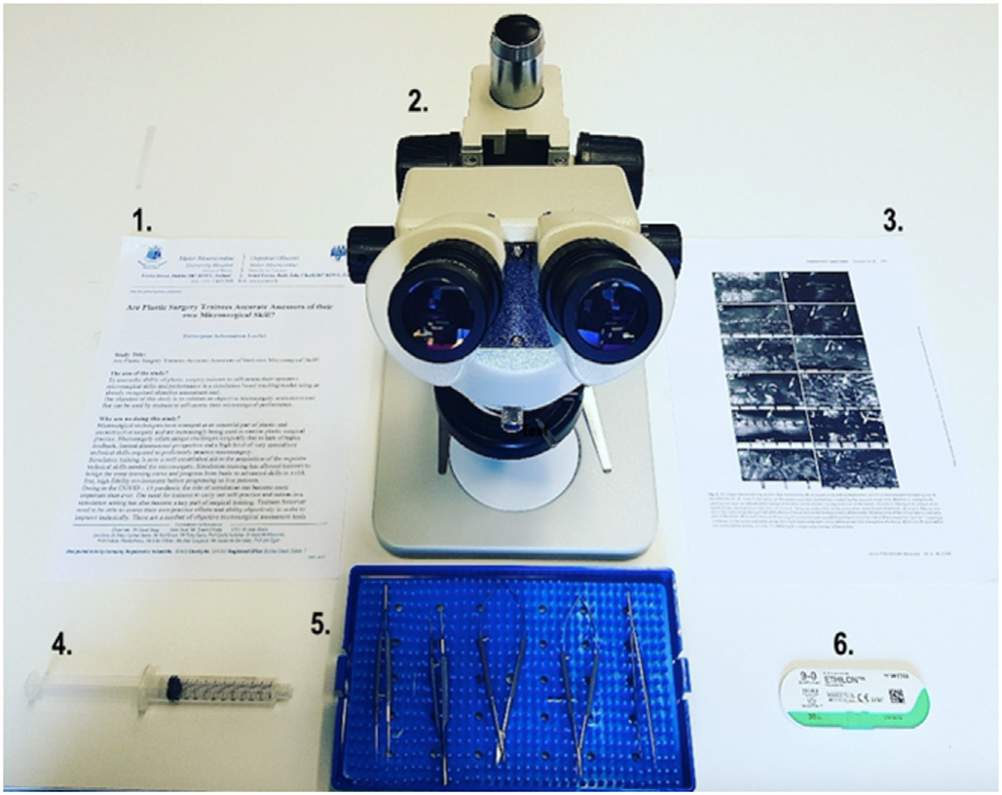
Standard microsurgical workstation. **1.** Participant Information Leaflet **2.** Desktop microscope with 45 × zoom **3.** Anastomosis Lapse Index Scoring Sheet **4.** 3.3 ml syringe with water **5**. Microsurgical instruments (micro-scissors, micro-needle holder, micro-forceps, and vessel dilator) **6.** 9.0 microsurgical suture.

**Figure 2:**
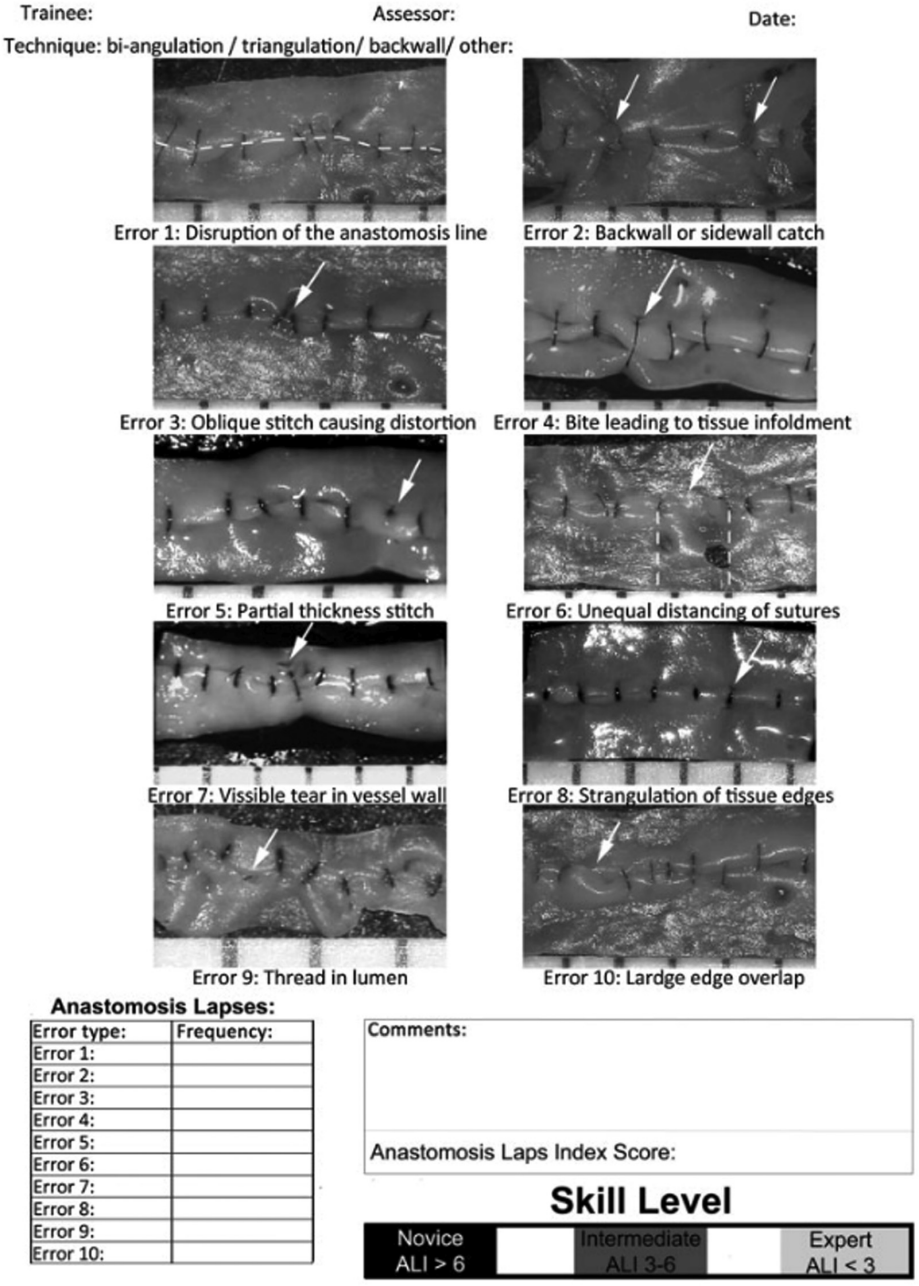
The Anastomosis Lapse Index – Ghanem et al^9^

Microsurgical workstations (Figure 1) were set up in the simulation laboratories at the Pillar Centre for Transformative Healthcare at MMUH and the Irish Centre for Applied Patient Safety and Simulation at GUH. Each station was equipped with a desktop surgical microscope (45 × zoom, 10 cm working distance), a microsurgical instrument kit (microsurgical needle holder, scissors, forceps, and vessel dilator), a 2 ml saline-filled syringe, and two 9.0 Ethilon sutures. Participants were familiarized with the testing procedure, and each participant was given the same written instructions to execute a well-defined task, which involved the application of eight interrupted microvascular sutures, evenly placed, around the chicken femoral vessel circumference, four sutures on the posterior wall, and four sutures on the anterior wall of the vessel, to result in an accurate microsurgical anastomosis.^2^ Participants were given 2 minutes to familiarize themselves with the surgical microscope and adjust it to their requirements. Participants were also familiarized with the ALI scoring system, and a magnified A3 copy of the scoring system was provided at each station (Figure 2).

**Figure 3:**
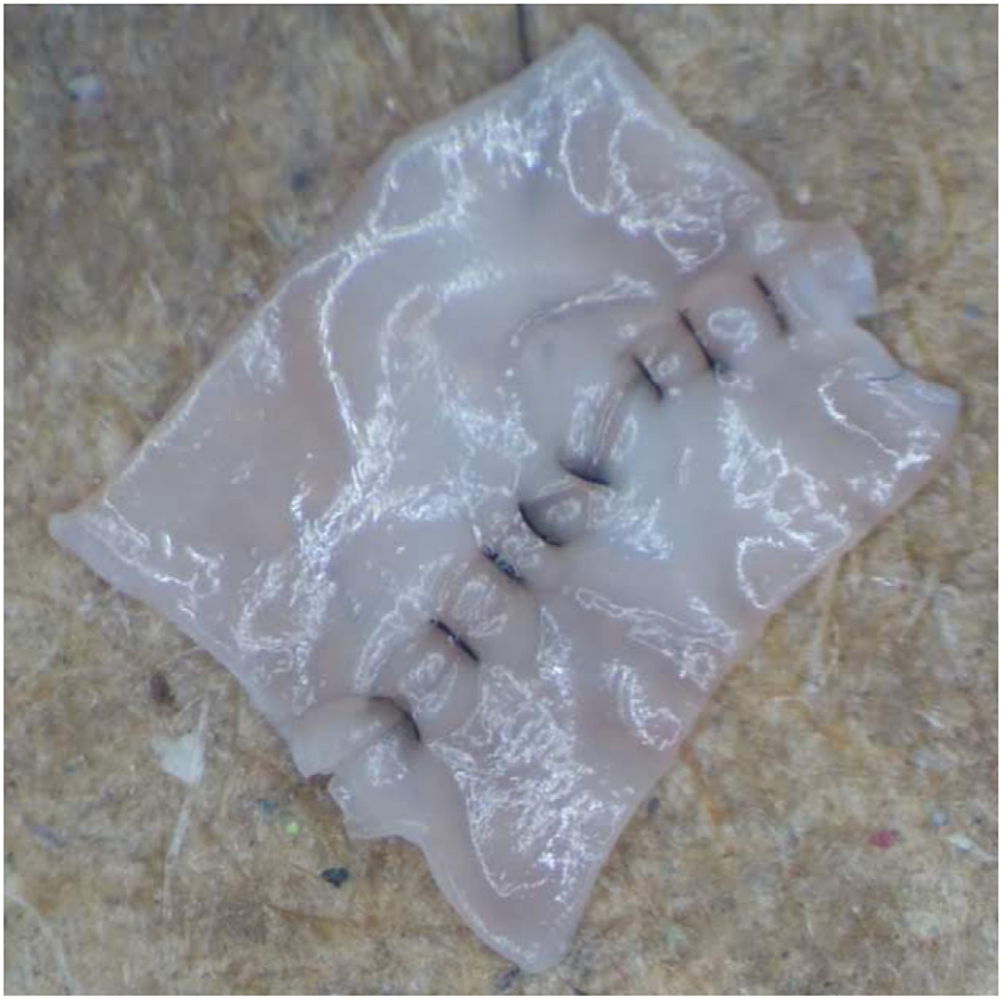
Example of micro-anastomosis with intimal surface exposed for scoring using the Anastomosis Lapse Index

Participants completed a microvascular anastomosis on the femoral artery of a pre-dissected, non-living chicken thigh model. The procedure was timed (time to completion [TTC]), with the timer started when the participant picked up the first surgical instrument and stopped when the final suture was completed. Upon completion, the vessel was cut longitudinally, and the intimal surface of the lumen was laid out and photographed at 25 × magnification, using a standardized procedure (Figure 3). A standardized photograph of the participant's anastomosis was uploaded to a laptop screen and the participant completed a self-assessment of the anastomosis using the ALI score to identify specific errors from the pre-determined list. The participant's ALI “self-score” was then recorded.

Anonymized anastomosis images were then scored independently by two expert consultant plastic surgeons, blinded to the participant's identity, level of expertise and self-score. The mean of the scores from the two consultant surgeons was recorded as the “expert-score.” Self-score and expert-score were compared to estimate the accuracy of the trainee self-assessment.

## Statistical Analysis

The data was initially plotted using distribution curves to assess for a normal distribution. A Shapiro–Wilk test was also carried out. The data was deemed to be non-parametric. Descriptive statistics were used to assess the demographic data collected from each participant. A Mann–Whitney U test was used to assess differences between males and females gender groupings about the self-score, expert-score, and TTC. A Mann–Whitney U test was also used to assess differences between novice and experienced trainees on all variables. A Wilcoxon signed-rank test was used to compare the self-score and expert-score assigned to each anastomosis. Significance was set at p=0.05 for all analysis conducted. All statistical analysis was carried out using the JASP statistical analysis package (JASP Team, 2018, Version 0.8.6).

## Results

### Demographic Data

**Figure 4:**
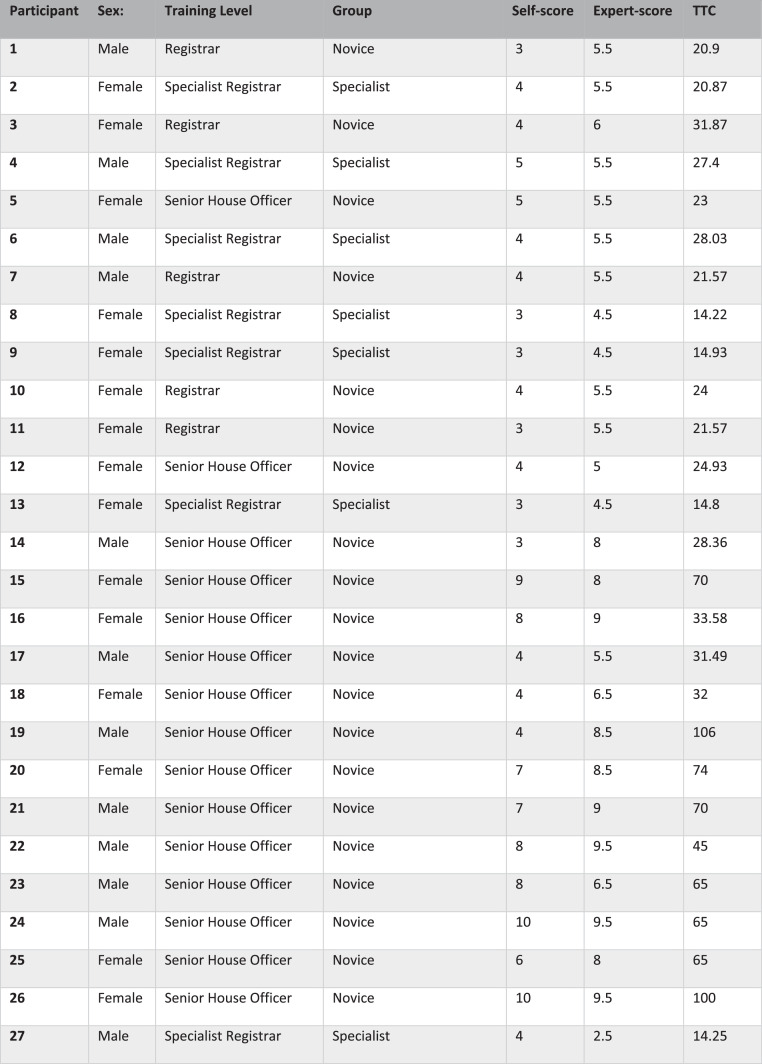
Demographic data and test data collected from all participants enrolled in the study TTC, time to completion

A total of 27 participants took part in the study (13 MMUH, 14 GUH). All participants had prior exposure to performing a microvascular anastomosis either in theater or at departmental simulation sessions. Fifteen were female and 12 were male. As per previous research from Tang et al; trainees were categorized according to microsurgical experience: 20 participants were categorized as “non-specialist,” while 7 were categorized as “specialist-less experienced.”^25^^,^^26^ For ease of reporting, these groups are referred to as “novice” and “specialist” hereafter. Full data for the cohort can be observed in Figure 4.

The median TTC for the entire cohort was 28.4 minutes (range 14.2–106.0). Within the specialist group, the median TTC was 14.9 minutes with a range of 14.2–28.0 minutes. Within the novice group, the median TTC was 32.8 minutes with a range of 20.9–106.0 minutes.

The median ALI self-score for the entire cohort was 4 (range 3–10), while the median ALI expert-score was 5.5 (range 2.5–9.5). The median self-score within the specialist group was 4.0 (range 3.0–5.0), while the median expert-score was 4.5 (range 2.5–5.5). The median self-score within the novice group was 4.5 (range 3.0–10.0), while the median expert-score was 7.25 (range 5.0–9.5).

### Self-score vs Expert-score

**Figure 5:**
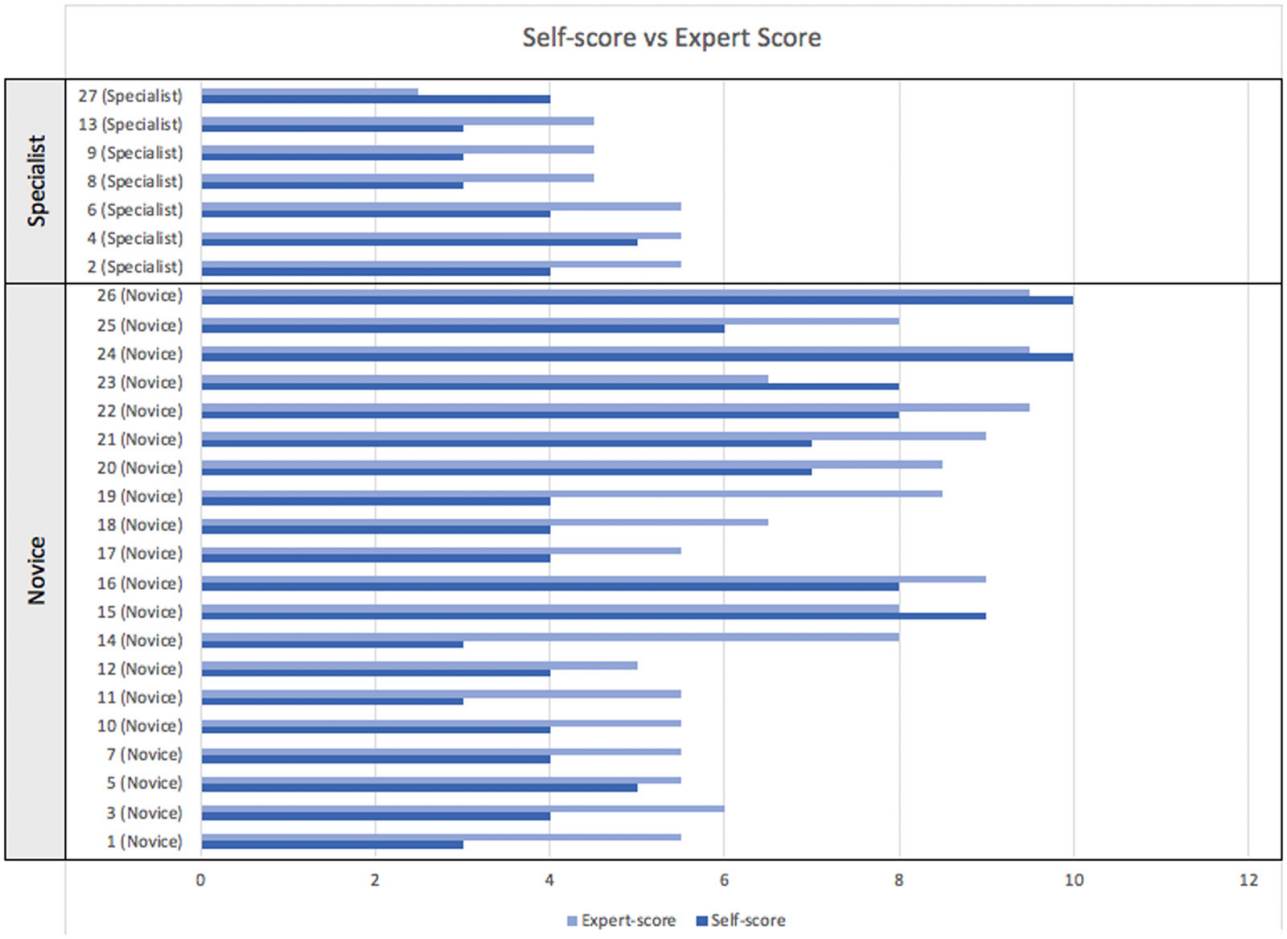
Chart comparing participant ALI self-score vs expert-score for microvascular anastomosis task

When data for the entire cohort (n=27) was analyzed, a Wilcoxon signed-rank test identified a significant difference between the trainee self-score and the expert-score (p<0.001, large effect size r=0.8) (Figure 5). Sub-analysis of the novice group (n = 20) demonstrated a significant difference between self-score and expert-score (p=0.001, large effect size: r=0.8), while analysis of the specialist group revealed no significant difference between self-score and expert-score.

### Subgroup Analysis

A Mann–Whitney U test identified a significant difference between the specialist group and the novice group for TTC (p=0.001, large effect size r=0.8), self-score (p=0.045, large effect size r=0.5) and expert-score (p=0.001, large effect size: r=0.8). A Mann–Whitney U test identified no significant difference between male and female trainees on any of the three outcome variables.

## Discussion

This study provides a robust contribution to the growing body of research in the field of microsurgery simulation training. Simulation training is a viable and cost-effective means of enabling trainees to obtain technical proficiency in a low-risk environment. Numerous high-fidelity supervised microsurgical simulation courses exist around the world. However, places on such courses are limited and for some trainees, cost is a limiting factor. Undoubtedly, these intensive hands-on instructive courses lead to the acquisition of microsurgical skills, however, it is also recognized that there is decay in such skills unless a trainee is practicing regularly. For these reasons, self-directed learning has become an increasingly important means of maintaining and improving skill levels. For self-directed learning to be effective, trainees must be able to accurately assess their skill level.

There is limited knowledge regarding the accuracy of trainee self-assessment of their technical skills. Several previous studies have explored the accuracy of surgical trainee self-assessment for macroscopic surgical techniques such as laparoscopy and endoscopy, with inconclusive results.^19^, ^20^, ^21^, ^22^, ^23^, ^24^ However, there is a significant paucity of research exploring the accuracy of trainee self-assessment of technical skills in the demanding field of microsurgery. To our knowledge, only one such study has been conducted, which demonstrated agreement between trainee self-assessment and independent expert assessment on a knot-tying and vessel anastomosis task.^27^ However, this study had a sample size of eight participants; therefore, further research with larger sample sizes is warranted to guide the future of microsurgical simulation training.

Another study by Arora et. al also demonstrated the ability of surgical trainees to accurately self-assess their skill when performing a laparoscopic procedure, regardless of their experience level.^20^ In contrast, we observed that across a cohort of plastic surgery trainees of varying experience levels, there was a significant difference between trainee self-assessment of their technical skill and that recorded by an independent expert assessor. In the vast majority of cases, there was a tendency for trainees to overestimate their technical skills. Interestingly, when a sub-analysis was conducted to explore the accuracy of trainee self-assessment within the specialist trainee group, there was no difference noted between the self-assessment score and that recorded by the expert assessors. This suggests that trainee self-assessment accuracy improves with experience. As microsurgery is a specialized field within surgery, it can be difficult for trainees to gain exposure to an adequate number of in-vivo microsurgical cases during their training. As a result, many trainees opt to seek microsurgical fellowships to gain the necessary experience. However, our research indicates that experienced trainees may be able to improve their technical skills and overcome the steep learning curve associated with microsurgery by engaging in independent simulation training, taking advantage of their ability to accurately self-assess.

Our study provides further evidence that the ALI score can accurately distinguish between novice and experienced trainees.^9^ In this study, specialist trainees performed the procedure significantly faster, with fewer self-assessed or expert-assessed errors than their novice counterparts. This suggests that the ALI score is a very useful tool for tracking trainee progress during microsurgical training programs. The ALI score has been shown to have excellent construct and predictive validity in non-living microvascular models, and given its simplicity and cost-efficacy, it is an extremely useful scoring model for trainees and trainers alike. Further research could investigate the ability of the ALI to grade performance in in-vivo microsurgical procedures to ascertain whether performance on non-living simulation models crosses over to real-life theater performance, although logistically and ethically this may prove challenging.

Although the ALI score is an objective and validated assessment tool, its objectivity is not absolute. Our research findings indicate that novice trainees are unable to accurately evaluate their technical skills using the ALI in the early stages of their training. Nevertheless, the results also demonstrate that this subjective bias decreases with experience. Despite novice trainees’ scoring of the ALI being subjective, regular usage and feedback from experts can assist trainees in developing a better understanding of their technical errors and identifying areas for improvement. This can be particularly beneficial for trainees to monitor their progress and self-assess their performance.

It is important to note that the assessment of technical skills should not solely rely on self-assessment, as it may be subject to biases or blind spots. Therefore, it is recommended that the self-assessment of trainees be supplemented with assessments from experts, who can provide a more objective evaluation and feedback on the trainee's technical skills. This is particularly relevant for novice trainees. In the era of remote working, this could be conducted virtually to minimize the disruption to service provision.

## Limitations

This study has several limitations. Our sample size comprised 27 trainees, with just 7 “specialist” trainees. This was limited by the number of surgical trainees employed by the Plastic and Reconstructive Surgery service across our two sites. Given that microsurgery is primarily the remit of Plastic and Reconstructive Surgeons, the decision was taken to exclusively include trainees on the Plastic and Reconstructive Surgery service with prior exposure to performing microsurgery. Future studies could be conducted across several sites to achieve larger sample sizes. Secondly, trainees were categorized according to microsurgical experience, using the Tang classification.^25^ While this approach is recommended in the literature,^26^ future studies could also document the number of microsurgical procedures completed by each trainee to more formally determine the trainee experience level.

## Conclusion

Simulation training provides an alternative to the traditional Halstedian surgical training model. It is an effective means of allowing surgical trainees to develop the technical skills required for surgery in a low-risk environment and will likely form a central role in microsurgical training in the future. Specialist trainees can accurately self-assess their technical skills and engage in independent self-directed microsurgical training and self-assessment. Novice trainees tend to overestimate their technical skills; therefore, expert feedback must be provided for novice trainees to facilitate individualized and targeted microsurgical simulation training.

## Conflicts of Interest

None
